# A Case Report of Atypical Presentation of Metastatic Breast Cancer as Peri-Bronchial Mediastinal Lesion

**DOI:** 10.7759/cureus.73794

**Published:** 2024-11-16

**Authors:** Sarah Tseu, Sadathulla Sharief, Syed Mehdi

**Affiliations:** 1 Geriatric Medicine, Lancashire Teaching Hospitals NHS Foundation Trust, Preston, GBR; 2 Pathology, Lancashire Teaching Hospitals NHS Foundation Trust, Preston, GBR; 3 Respiratory Medicine, Lancashire Teaching Hospitals NHS Foundation Trust, Preston, GBR

**Keywords:** breast cancer, endobronchial ultrasound (ebus), esophageal stricture, late recurrence breast cancer, mediastinal lesion, progressive dysphagia

## Abstract

Breast cancer is the most prevalent cancer among women worldwide. Despite significant advancements in breast cancer treatments over the past decade, late recurrence, a hallmark of breast cancer, remains a major challenge for oncologists. In this case report, we present an atypical presentation of late breast cancer recurrence as a peri-bronchial lesion manifesting as dysphagia 14 years after completing treatment for primary breast cancer. Radiological imaging revealed a peri-bronchial lesion and a subcutaneous nodule in the left mastectomy bed. This case posed a diagnostic dilemma for the attending physicians in distinguishing between late breast cancer recurrence and the possibility of two synchronous primary malignancies. The patient was ultimately diagnosed with metastatic breast cancer with histological confirmation. This case underscores the importance of correlating clinical presentation, radiological images, histopathology examinations and immunohistochemical results to ensure a comprehensive and accurate diagnosis.

## Introduction

Breast cancer is the most common cancer in women in the United Kingdom (UK) [1]. There are approximately 56,800 new diagnoses of breast cancer in the UK annually [1]. A breast lump is the most common presenting symptom, followed by nipple abnormalities, mastalgia, and cutaneous changes [2]. The overall prognosis for breast cancer is generally good, with a 10-year breast cancer survival rate of 75.9% [1]. Breast cancer treatment depends on histology subtype and staging and is mainly comprised of surgery, chemotherapy, radiotherapy, hormonal therapy, and immunotherapy [3].

Breast cancer is considered a micrometastatic disease, with micrometastases potentially occurring prior to primary breast cancer detection [4]. After primary therapy, these micrometastatic cells can remain dormant for decades [4]. Alteration in the tumor environment can reverse tumor dormancy and trigger proliferation, which aligns with the characteristic late recurrence commonly observed in breast cancer [4,5].

## Case presentation

We present the case of a 58-year-old woman who presented to the emergency department with a three-month history of epigastric pain associated with a one-month history of nausea, vomiting, and progressively worsening dysphagia. Her dysphagia was initially limited to solids but progressed to involve liquids within three months. The patient also reported loss of appetite with unintentional weight loss of 10 kg over four months.

Her past medical history included gastroesophageal reflux disease and T1N3M0 grade two invasive ductal carcinoma of the left breast, which was diagnosed and treated 14 years ago. She had undergone a left mastectomy and adjuvant chemoradiotherapy therapy with docetaxel and epirubicin. She also received tamoxifen for five years. She resided with her family and was independent in her daily activities. Her Eastern Cooperative Oncology Group (ECOG) performance status was zero. She was an ex-smoker with an 18-pack-year history.

The patient was initially admitted for rehydration. Blood tests, electrocardiogram, and chest X-ray were unremarkable. An urgent oesophagogastroduodenoscopy (OGD) demonstrated a tight lower esophageal stricture, an appearance in keeping with probable peptic stricture with no evidence of cancer. Esophageal stricture biopsies showed no significant abnormality. Although the esophageal stricture was amenable to dilatation, a computed tomography (CT) scan of the thorax was recommended by the gastroenterologist to look for extrinsic compression, given a past history of cancer.

**Figure 1:**
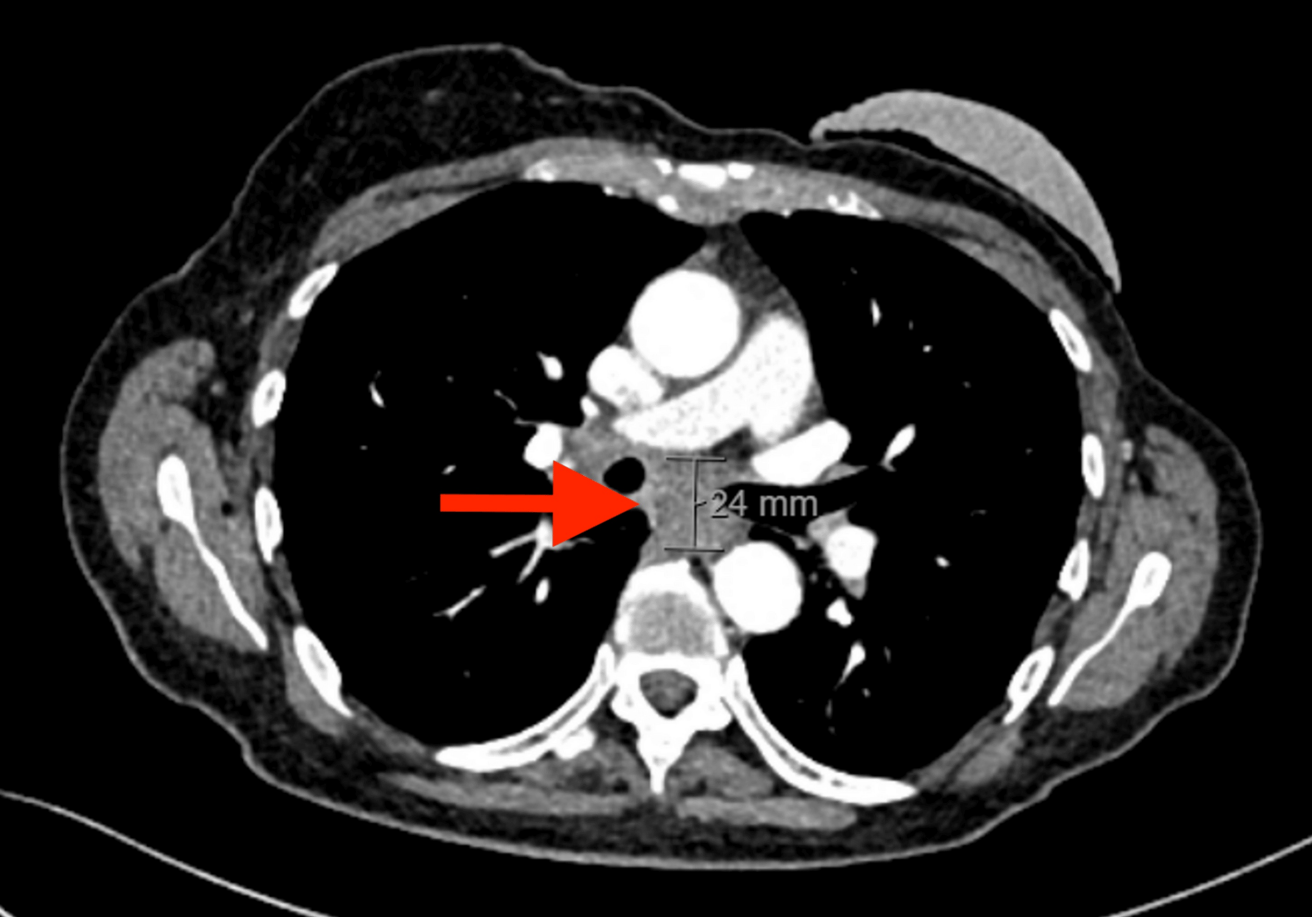
CT thorax with contrast revealed a peri-bronchial mediastinal soft tissue lesion causing stenosis in the middle esophagus (red arrow). CT: computed tomography.

A CT thorax with contrast revealed an extraluminal soft tissue surrounding the esophagus at the subcarinal region. It extended along the peri-bronchial region of the right main bronchus, with appearances concerning malignant infiltration (Figure 1).

She was readmitted a month later with abdominal pain, progressive dysphagia, and persistent vomiting. An urgent OGD was performed, and the esophageal stricture was successfully dilated.

**Figure 2:**
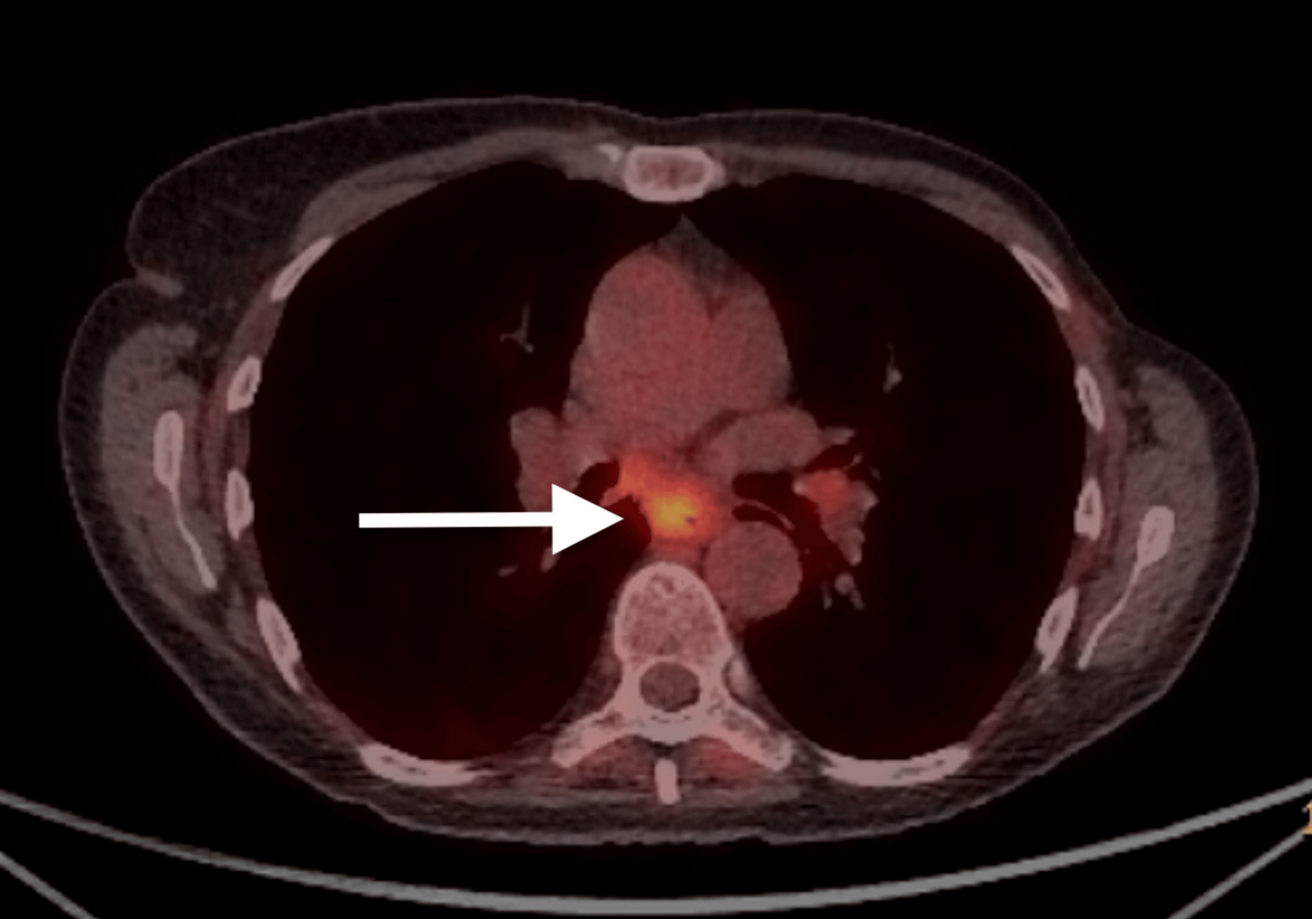
PET scan demonstrating soft tissue infiltration at the level of the carina, which was inseparable from the esophagus, with high FDG uptake (white arrow). PET: positron emission tomography, FDG: fluorodeoxyglucose.

**Figure 3:**
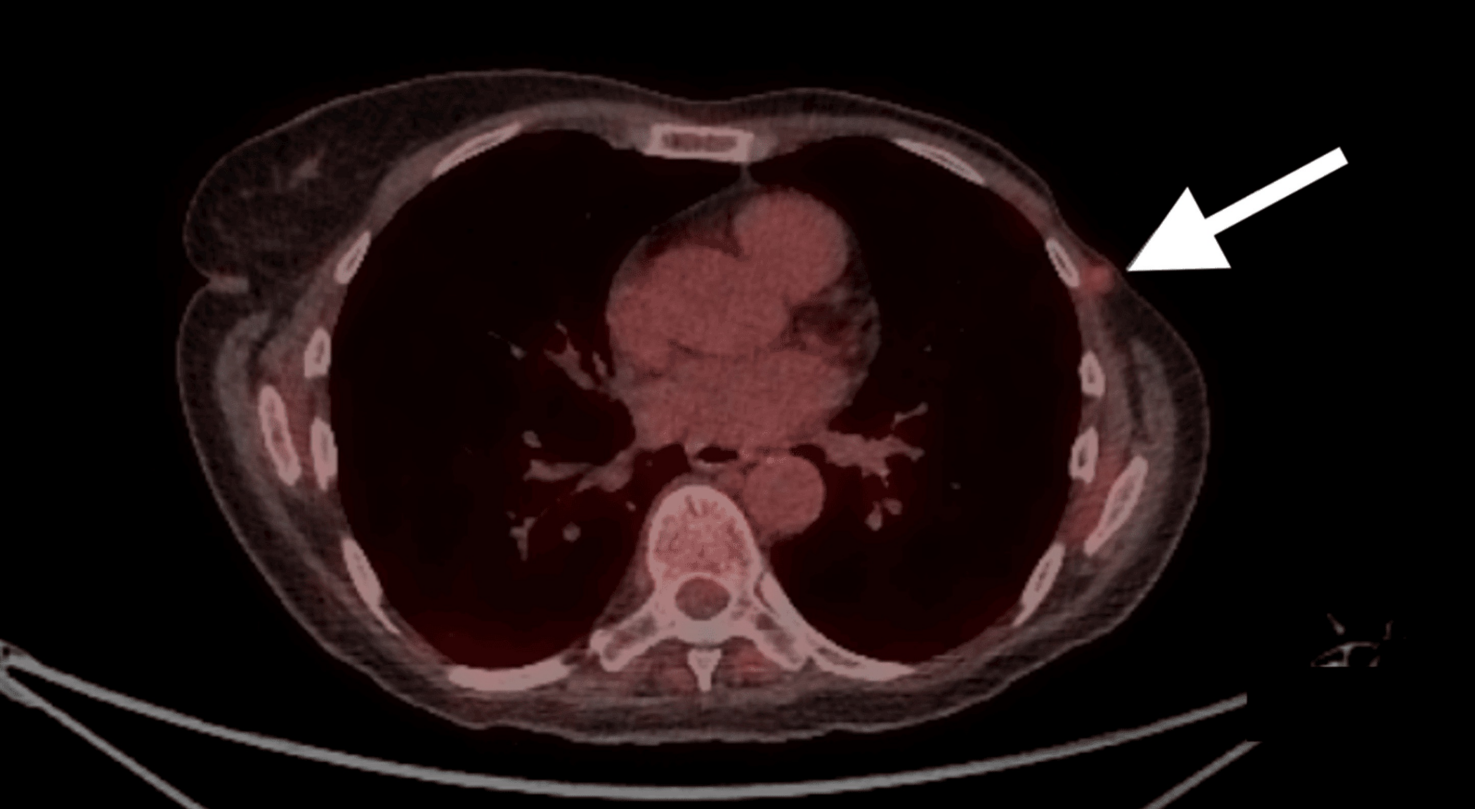
Arrow pointing at subcutaneous soft tissue nodule (0.7 cm) at left mastectomy bed, exhibiting low FDG uptake in PET scan (white arrow). PET: positron emission tomography, FDG: fluorodeoxyglucose.

The patient then had an outpatient positron emission tomography (PET) scan reported as below: I. A soft tissue lesion at the level of the carina, inseparable from the esophagus, exhibiting high fluorodeoxyglucose (FDG) uptake (Figure 2). II. A subcutaneous nodule in the left mastectomy bed with low FDG uptake (Figure 3).

**Figure 4:**
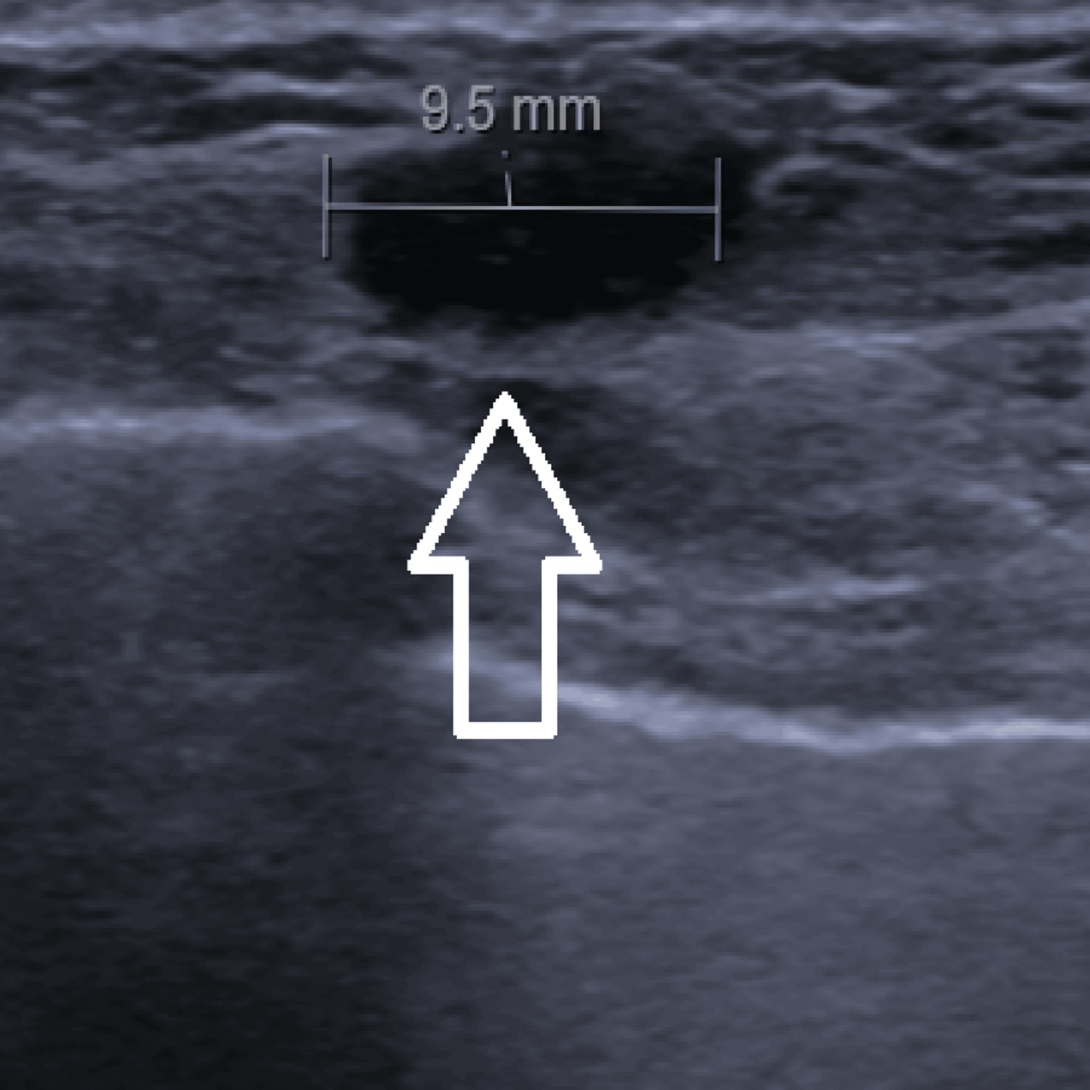
Ultrasound of breast demonstrating 9.5 mm nodule at the edge of left mastectomy scar (white arrow).

She subsequently had an urgent outpatient breast ultrasound, revealing a 9.5 mm nodule at the edge of the left mastectomy scar (Figure 4). Cytology from fine needle aspiration (FNA) was consistent with recurrent breast carcinoma.

**Figure 5:**
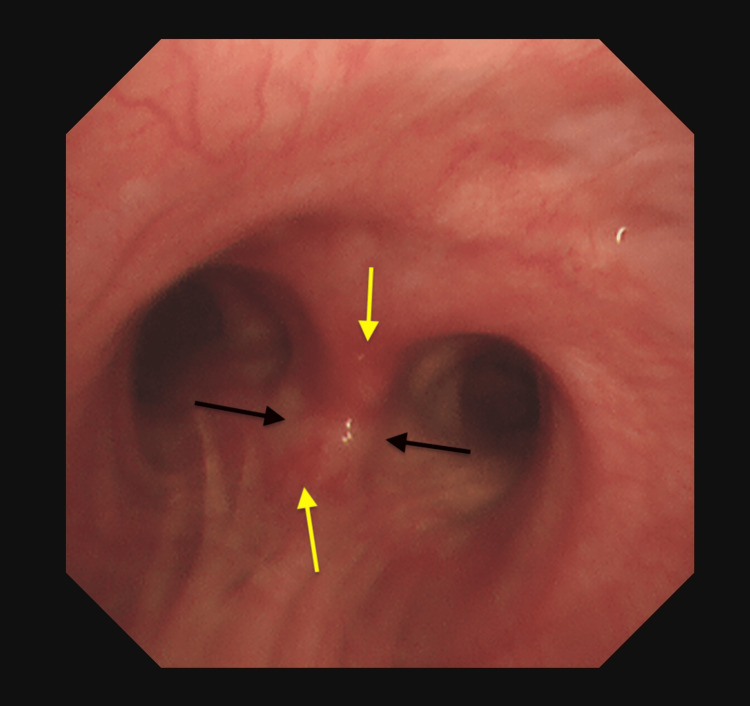
Bronchoscopy showed splayed carina (black arrows) with increased submucosal vascularity (yellow arrows).

**Figure 6:**
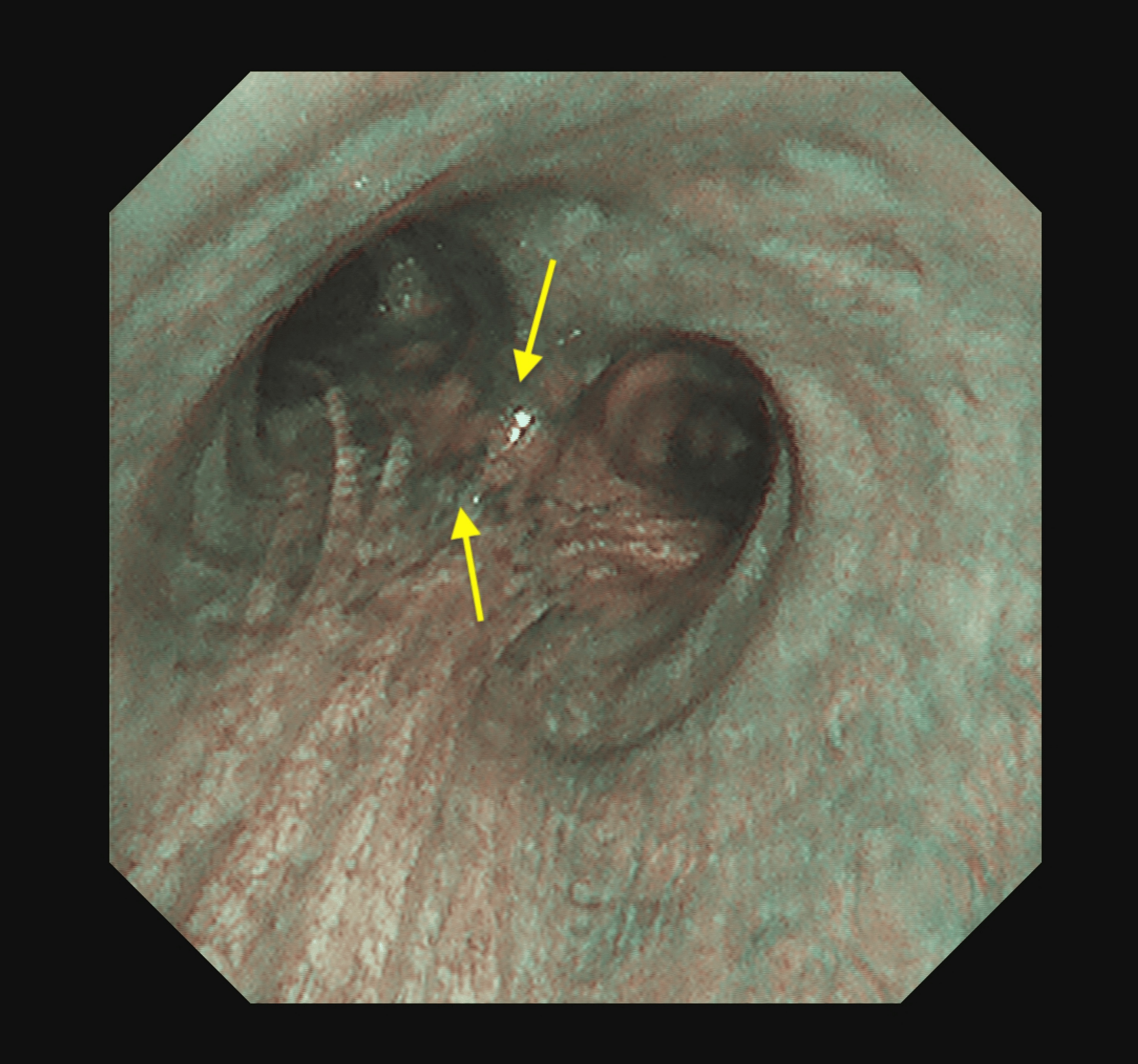
Submucosal vascularity (black staining, indicated by yellow arrows) is more prominent in a specialized mode of bronchoscopy.

**Figure 7:**
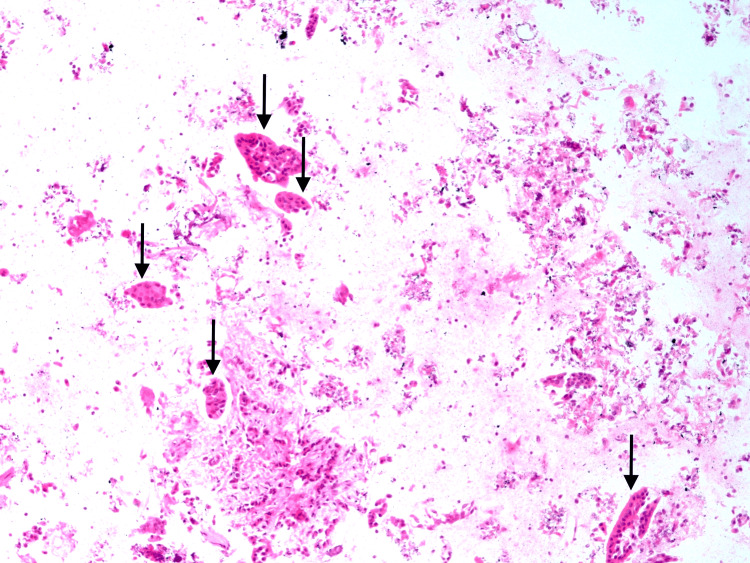
Malignant epithelial cells in clusters and infiltrating cords (black arrows).

**Figure 8:**
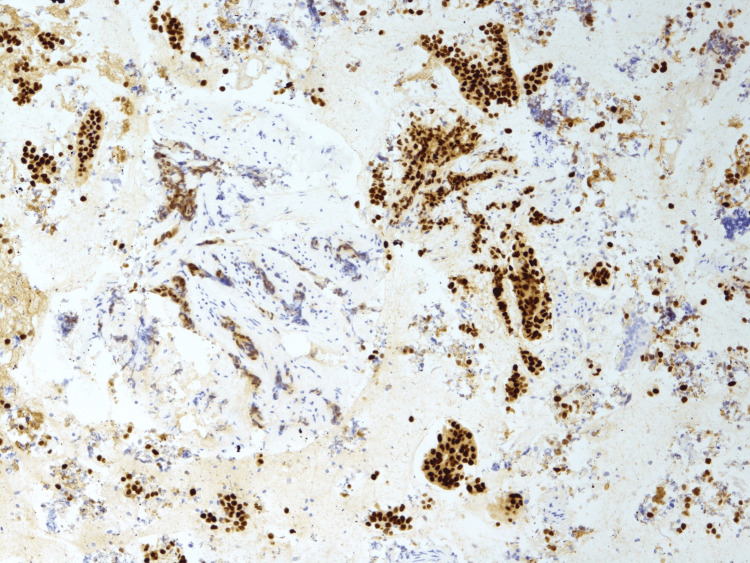
Malignant cells positive with estrogen receptors (brown stained).

Endobronchial ultrasound (EBUS) and bronchoscopy identified a large 3 cm oval-shaped subcarinal lymph node with homogenous blood vessels (Figures 5, 6). The lymph node was successfully biopsied. The EBUS transbronchial needle aspiration (TBNA) histology was in concordance with metastatic breast cancer, estrogen receptors (ERs) positive 8/8, and human epidermal growth factor receptor 2 (HER2) negative (Figures 7, 8). These malignant cells were positive with GATA binding protein 3 (GATA-3) and negative with thyroid transcription factor 1 (TTF-1) and P40 immunohistochemistry. Immunohistochemistry test result relates to previous breast cancer.

The patient was diagnosed with metastatic breast cancer in the subcarinal nodes and recurrent nodules in the left mastectomy scar. The patient was followed up in the oncology clinic two months later. During this visit, she was consented to start treatment with palliative intent. She was commenced on endocrine therapy, letrozole, indefinitely and cyclin-dependent kinases (CDK) 4/6 inhibitor, abemaciclib. Abemaciclib was preferred over palbociclib as it comes in a smaller tablet, considering ease of swallowing.

## Discussion

The primary diagnostic dilemma in this case report lies in distinguishing between metastatic breast cancer and the possibility of two synchronous primary malignancies, specifically late recurrence of breast carcinoma in the left mastectomy bed and lung carcinoma. It is highly atypical for breast cancer to manifest as a subcarinal lymph node, leading to symptoms such as dysphagia. This uncommon presentation adds complexity to the diagnostic process and highlights the importance of histological diagnosis from multiple sites, as relying solely on anatomical evaluation for cancer diagnosis can lead to erroneous conclusions.

As observed in the study conducted by Pedersen et al., the cumulative incidence of breast cancer recurrence during follow-up was 17% among 20,315 patients who were disease-free at 10 years [4]. Micrometastatic cells may remain inactive for decades, with case reports of late breast cancer recurrence occurring as long as three decades after initial diagnosis [4]. The risk of late recurrence of breast cancer is higher in those with high nodal burden, large tumor size, and ER-positivity [4]. Distant recurrences, such as soft tissue, distant nodal involvement, and bone and visceral metastases, are more common in the ER-positive subgroup compared to the ER-negative subgroup [6]. 

In concordance with 2018 National Institute for Health and Care Excellence (NICE) guidelines, post-menopausal women with ER-positive invasive breast cancer should be offered extended endocrine therapy with an aromatase inhibitor after completing initial five years of tamoxifen therapy, provided the treatment was tolerated [7]. Evidence suggests lower rates of late breast cancer recurrence in those with extended endocrine therapy [7].

In the PALOMA-2 study conducted by Finn et al., there is strong evidence of significantly longer progression-free survival amongst post-menopausal women with ER-positive, HER2-negative advanced breast cancer in those who received combined treatment with endocrine therapy and CDK 4/6 inhibitor compared to those who only received endocrine therapy [8].

CDK inhibitors serve as a negative regulator of cell cycle progression by hindering the hyperphosphorylation of the retinoblastoma gene product, thereby halting progression to the S phase of the cell cycle [8]. Myelotoxic effects, including leukopenia, anemia, and neutropenia, are common side effects of CDK inhibitors [8]. Patients experiencing these adverse effects benefit from supportive management and dose reduction [8].

The National Comprehensive Cancer Network (NCCN) recommends that patients with invasive breast cancer receive routine history and examinations, along with annual mammography, as part of surveillance protocol [9]. During follow-up, oncologists should assess adherence to adjuvant endocrine therapy and encourage compliance to help reduce the risk of late breast cancer recurrence [9]. 

As breast cancer survival rates continue to improve due to advancements in medical treatments, late recurrence has emerged as a significant challenge for physicians. Numerous validation tools are available to identify patients at high risk of late breast cancer recurrence. However, there is limited literature and guidance on surveillance strategies for patients with a high risk of late recurrence of breast cancer. 

## Conclusions

Breast cancer is the most common cancer among women. The unique characteristics of breast cancer include specific cell expression, tumor dormancy leading to late recurrence and micrometastases. It is essential to integrate clinical presentation, radiological images, histopathology examinations, and immunohistochemical results when diagnosing a new case of cancer. This ensures a more accurate diagnosis, which is critical to optimize personalized oncological treatments. Due to the nature of late breast cancer recurrence, it is crucial for physicians to maintain a low index of suspicion to promptly identify possible recurrence in long-term survivors.
